# Correlation Between Propulsive Velocity, Maximum Velocity, and Power and the 2D:4D Ratio in Paralympic Powerlifting Athletes

**DOI:** 10.1155/bmri/8701719

**Published:** 2025-02-28

**Authors:** Álvaro Fontes da Silva Neto, Felipe J. Aidar, Ângelo de Almeida Paz, Jymmys L. Santos, Raphael Fabricio de Souza, Paulo Francisco de Almeida-Neto, Koulla Parpa, Breno Guilherme de Araújo Tinoco Cabral, Anderson Carlos Marçal, Georgian Badicu

**Affiliations:** ^1^Graduate Program of Movement Science, Federal University of Sergipe (UFS), São Cristovão, Brazil; ^2^Group of Studies and Research of Performance, Sport, Health and Paralympic Sports (GEPEPS), Federal University of Sergipe (UFS), São Cristovão, Brazil; ^3^Department of Physical Education, Federal University of Sergipe (UFS), São Cristovão, Brazil; ^4^Graduate Program of Physiological Science, Federal University of Sergipe (UFS), São Cristovão, Brazil; ^5^Department of Physical Education, Federal University of Rio Grande do Norte, Natal, Brazil; ^6^University of Central Lancashire, Cyprus Campus, Pyla, Cyprus; ^7^Department of Physical Education and Special Motricity, Transilvania University of Brasov, Brasov, Romania

**Keywords:** Paralympic powerlifting, performance predictor, prenatal testosterone

## Abstract

**Background:** Among the strength sports we have Paralympic powerlifting, and the factors that influence strength have been investigated; among them is the relationship between strength and the ratio of the size of the second and fourth fingers of the hand (2D:4D).

**Objectives:** The study is aimed at evaluating the relationship between the 2D:4D finger length ratio and the dynamic strength indicators, mean propulsive velocity (MPV), maximum velocity (Vmax), and power, with loads of 45% and 80% of one repetition maximum (1RM), in Paralympic powerlifting.

**Methodology:** Sixteen elite Paralympic powerlifting athletes were evaluated for dynamic strength indicators, MPV, Vmax, and power, with loads of 45% and 80% 1RM. The 2D:4D proportions and correlations between the indicators were evaluated of 2D:2D ratios and dynamic strength indicators.

**Results:** Moderate correlations were found between MPV 45% and 4D (*r* = 0.551, *p* = 0.027), between MPV 45% and R-L 2D:4D diff. (*r* = −0.595, *p* = 0.015), between power 80% and L2 (*r* = 0.542, *p* = 0.030), and between MPV 45% and R-L 2D:4D (*r* = −0.585, *p* = 0.017). There was also a moderate correlation between power 80% left 2D (*r* = −0.542, *p* = 0.030). However, no correlation was found between the 2D:4D ratios and dynamic strength indicators in Paralympic powerlifting.

**Conclusion:** The 2D:4D ratio presents a moderate correlation with dynamic strength indicators in Paralympic powerlifting athletes, where the ratios with the velocity of 45 of 1RM can be used as a predictor but with caution.

## 1. Introduction

Powerlifting is a strength sport where both conventional powerlifting (CP) and Paralympic powerlifting (PP) athletes compete to lift the heaviest loads [1–3]. Powerlifting training typically employs free weights with high loads and intensities [4], and strength training has been the subject of research related to physiological parameters [4–7]. Several studies have correlated muscular strength with hormonal factors and testosterone levels [8–10].

In this context, the 2D:4D digit ratio has been proposed as a marker of prenatal testosterone exposure and the testosterone–estradiol ratio. The ratio between the second and fourth digits (2D:4D) is hypothesized to be lower in individuals with higher prenatal testosterone exposure, resulting in either a correlation between 2D:4D and muscular strength depending on sex [11–16].

The 2D:4D ratio (R2D:4D) has been associated with athletic performance [17, 18], particularly in strength and speed sports [19, 20]. Some studies have demonstrated a negative correlation between the R2D:4D and performance [12, 21–24], while others have shown a positive relationship [25–27].

The R2D:4D and testosterone were predictors of strength manifestation through countermovement jump (CMJ) height. It was also observed that testosterone concentration and R2D:4D were predictors of CMJ in young athletes [28]. Similarly, the R2D:4D was shown to be a good predictor of physical fitness in soccer players, and the R2D:4D would be a good predictor in identifying talent and the performance of young soccer players [18]. In the same direction, it was suggested that regardless of gender, college tennis athletes have lower 2D:4D values than nonathletes, where 2D:4D would be a good predictor of performance [29]. Still addressing what could interfere in the 2D:4D relationship and performance, a set of variables was observed that can influence the athlete's development and the acquisition of sports skills, among them the identification and development of talents, interest in sports practice, and the implication of talents related to training [30]. Thus, it would not be enough to have aptitude, in this case issues related to prenatal testosterone. But there would also be intervention in performance in relation to the quantity and quality of training [31] or even psychological factors, such as motivation and resilience [30].

However, other studies have not found a clear relationship between the R2D:4D and performance in Paralympic athletes [32]. Additionally, it has been observed that PP athletes may exhibit greater muscular strength compared to conventional athletes [2, 3].

Given this background, the objective of our study was to evaluate the relationship between the R2D:4D and performance, as well as dynamic indicators of strength, mean propulsive velocity (MPV), maximum velocity (Vmax), and power, with loads of 45% and 80% of one repetition maximum (1RM), in PP. We hypothesize that there is a positive relationship between the R2D:4D and speed and power performance in Paralympic weightlifting and that factors such as training could interfere with this relationship.

## 2. Methodology

### 2.1. Study Design

**Figure 1:**
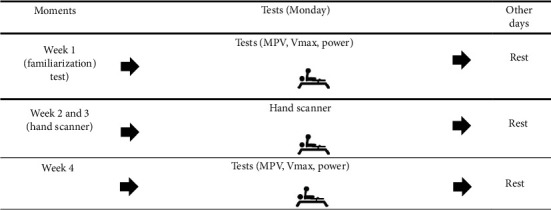
Experimental study design.

All procedures were conducted at the same time (09:00–12:00) for each participant under consistent environmental conditions (air temperature between 22°C and 26°C and relative humidity around 60%) in a weight training room at the Federal University of Sergipe. During the first week, participants underwent a familiarization session. From the second week onwards, data collection took place between 08:00 and 12:00, according to the participants' availability. The second and fourth weeks were dedicated to scanning the athletes' hands, while the third week focused on collecting dynamic strength indicators. Participants were familiarized with the procedures and rested for at least 48 h before the experiments. They were instructed to maintain their usual routines during the evaluation days, avoiding strenuous exercise and abstaining from caffeine consumption 48 h before the test (Figure 1).

### 2.2. Participants

The sample for this study consisted of 16 male individuals aged between 18 and 40 years. These athletes had over 24 months of experience in PP training and competitive experience at the national level, all eligible for the modality according to the rules of the International Paralympic Committee [33] and ranked among the top 10 in their respective categories. Six athletes had lower limb malformations (arthrogryposis), three had sequelae of poliomyelitis, five were amputees, and two had spinal cord injuries (below T8) due to trauma. G⁣^∗^Power software (University of Düsseldorf, Düsseldorf, Germany) was used to perform sample calculations. A power of 0.8 and a high effect size were used; this was associated with another study that adopted a similar sample [32, 34].

Previous studies have reported high to very high correlations between physical conditioning parameters and training load, as well as the R2D:4D [4, 35, 36]. Therefore, the results were analyzed to obtain a sample size with at least 80% power. The variables considered in the analysis were two-tailed, with an *α* error of < 0.05 and a very large effect size. All participants were volunteers and signed the informed consent form in accordance with Resolution 466/2012 of the National Health Council's National Research Ethics Commission (CONEP). We also adhered to the ethical principles expressed in the Declaration of Helsinki 1964, revised in 2013 of the World Medical Association. This study was approved by the Research Ethics Committee of the Federal University of Sergipe, Approval Number CAAE: 67953622.7000.5546, Technical Opinion 6.523.247 (approval date November 22, 2023). The sample characteristics are displayed in Table 1.

### 2.3. Instruments and Procedures

The body mass of athletes was measured using a digital wheelchair scale with an electronic platform, where the athletes were assessed in a seated position (Micheletti, São Paulo, Brazil). The scale has a maximum weight capacity of 300 kg and dimensions of 1.50 by 1.50 m. The bench press exercise was performed on an official bench (2.10 m in length, Eleiko, Halmstad, Sweden) and an Olympic barbell (220 cm in total length, 20 kg weight) approved by the IPC [38].

#### 2.3.1. Dynamic Force Indicators

**Figure 2:**
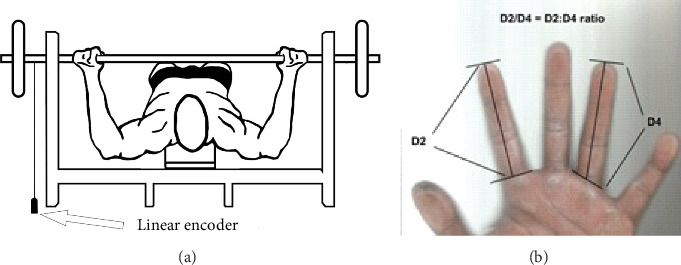
Demonstration of the positioning of the linear encoder for evaluation of dynamic force indicators (a) and the fingers' proportion model 2D:4D (b).

The dynamic strength parameters, MPV, Vmax, and power, were recorded using a Speed4Lift encoder (Vitruve, Madrid, Spain) [39]. The analysis of these parameters was performed using a load of 45% of 1RM, where the velocity was approximately 1.0 m·s^−1^ [40, 41]. A load of 80% of 1RM was also used, given that this is normally the load used in strength training [42]. Data collection began with a warm-up consisting of a 10-min session that included 20 repetitions of shoulder abduction with dumbbells, shoulder presses, and shoulder rotations with dumbbells. Subsequently, a specific warm-up on the bench press was performed with the bar weight (20 kg) and 10 slow repetitions (3.0 × 1.0 s, eccentric × concentric) followed by 10 fast repetitions (1.0 × 1.0 s, eccentric × concentric). The athletes then performed a set of four repetitions at 45% and 80% 1RM at the maximum possible velocity [32, 42, 43], as well as measurements of finger lengths and their ratio [32] (Figure 2).

#### 2.3.2. Finger Measurements

The measurement of the 2D and 4D finger lengths followed a previously established protocol [14, 32]. Athletes were instructed to place their right and left palms on a scanner, with fingers positioned approximately 2.0 cm apart. The scanned palm images were transferred to Kinovea Software Version 0.9.5 (Free Software Foundation Inc., Boston, Massachusetts, United States) for finger length measurement.

The length of the 2D and 4D fingers was measured from the basal proximal phalange (flexion) to the distal phalange. The ratio of both fingers was calculated by dividing 2D by 4D. A Brother Scanner (Brother Industries, Nagoya, Japan) was used, with a measurement precision of 0.01 cm for the second and fourth fingers to the fingertip. The difference between the right hand 2D:4D ratio (RF2D:4D) and the left hand 2D:4D ratio (LF2D:4D) was calculated [18, 25].

**Figure 3:**
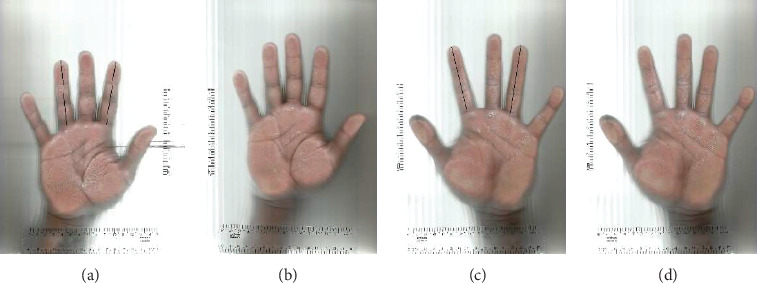
(a–d) Representative hand scanner with markings.

All assessments were conducted by the same evaluator, and intraobserver reliability was assessed twice, with measurements taken 1 week apart. The intraclass correlation coefficient (ICC) for the R2D:4D ranged from 0.95 to 0.99.  Figure 3 provides an example of the finger length capture and measurement process.

#### 2.3.3. Analysis

All procedures were conducted within a consistent time frame (09:00–12:00) for each participant under standardized environmental conditions (ambient temperature between 23°C and 25°C, relative humidity approximately 60%) in a weight training facility at the Federal University of Sergipe. During the initial week, subjects underwent a familiarization session. From the second week onward, data collection was performed between 09:00 and 12:00, accommodating participants' schedules. For the assessment of MPV, Vmax, and power, participants were instructed to execute four repetitions [42].

### 2.4. Statistics

Descriptive statistics were performed using measures of central tendency, including mean ± standard deviation (*X* ± SD) and 95% confidence intervals (95% CIs). The Shapiro–Wilk test was used to assess the normality of variables, considering the sample size.

The a priori model was considered in accordance with the main objective of the study: *t*-test correlation: point biserial model [44, 45]. Correlation coefficient (*r*) thresholds were defined as > 0.1 = trivial, 0.1–0.3 = small, 0.3–0.5 = moderate, 0.5–0.7 = large, 0.7–0.9 = very large, and > 0.9 = nearly perfect [46], and *R*^2^ was still used as the coefficient of determination. Statistical analyses were conducted using IBM SPSS Statistics Version 25.0 (IBM, New York, United States).

## 3. Results

Table 2 contains the descriptive values of finger length and the respective relationships discussed and the dynamic strength indicators.

Table 3 contains the results of the correlations of finger relations and dynamic force indicators.

There was a correlation between MPV 45% and right fourth, *r* = 0.551 (moderate correlation), *p* = 0.027, *R*^2^ = 0.30 (explaining 30% of the phenomenon). MVP 45% also had a negative correlation with right 2D:4D, *r* = −0.595 (moderate correlation), *p* = 0.015, and *R*^2^ = 0.35 (explaining 35% of the phenomenon), and there was also a correlation between MVP 45% and R-L 2D:4D diff., *r* = −0.585, *p* = 0.017, *R*^2^ = 0.34 (explaining 34% of the phenomenon). There was also a correlation between power 80% left 2D, *r* = −0.542 (moderate correlation), *p* = 0.030, *R*^2^ = 0.29 (explaining 29% of the phenomenon).

## 4. Discussion

The objective of our study was to evaluate the relationship between the 2D:4D digit ratio and performance metrics (MPV, Vmax, and power) in PP.

In our study, similar to previous research, we observed a relationship between the RF2D:4D and dynamic strength indicators. We also found a relationship between the LF2D:4D and power indicators [29, 31, 47]. Considering that studies indicate the right hand shows a stronger relationship than the left hand, suggesting the right hand is more representative of prenatal androgenic influence [48], our study confirmed this more evident relationship with the right hand, although the left hand showed a better relationship with power.

The results of the present study reveal a significant positive correlation between the length of the fourth finger of the right hand (R4) and MPV, as indicated by a moderate Pearson correlation coefficient. These findings are consistent with the existing literature, suggesting that specific physical characteristics, such as finger length, may be related to athletic performance. The positive linear relationship found indicates that as the length of the right index finger increases, there is a corresponding tendency for an increase in MPV. This finding can be explained by the fact that the R2D:4D, which involves the length of the second and fourth fingers, is frequently used as a marker of prenatal androgen exposure [49–52]. Previous studies demonstrate that a lower R2D:4D is associated with greater androgen exposure during fetal development, which can influence physical characteristics and athletic capabilities in adulthood [53, 54].

Prenatal testosterone is associated with various genes and their development, influencing the growth and development of multiple systems, particularly the musculoskeletal system [51, 52]. Consequently, additional exposure to testosterone during the prenatal phase tends to produce positive effects on force generation capacity. Furthermore, prenatal testosterone stimulates the endocrine system, providing an immediate response in relation to testosterone peaks [49, 50].

Prenatal testosterone tends to stimulate the endocrine system, resulting in short-term responses associated with testosterone peaks [49, 50]. These responses are observed in situations requiring aggressive behavior in challenging circumstances, with vigorous responses to intensive training or competition [50, 55, 56]. Although our study found positive correlations, it is important to note that in some studies, the R2D:4D showed a negative correlation with strength, independent of variables such as body size, hormone levels, and aggressiveness [57, 58].

Manning et al. [54, 59] reported that individuals with a lower R2D:4D, often correlated with a longer index finger relative to the ring finger, tend to exhibit better performance in physical activities requiring strength and velocity. Hönekopp et al. [53] observed that this relationship may be more pronounced in males, suggesting a differential androgenic influence based on sex.

Complementing these findings, we observed a significant negative correlation between the RF2D:4D and MPV, with a moderate negative Pearson correlation coefficient. This result indicates that a lower R2D:4D is associated with a higher MPV, suggesting that prenatal exposure to higher levels of testosterone may be related to greater capacity for velocity and muscular explosiveness [54, 60].

Furthermore, the difference between the right and left hand 2D:4D ratios (R-L 2D:4D) demonstrated a significant negative correlation with MPV, yielding a moderate negative Pearson correlation coefficient. This finding suggests that a greater disparity between the R2D:4D of the hands is associated with lower MPV, potentially indicating a more pronounced influence of biological factors on the dominant hand, thereby affecting physical performance [29, 31, 47, 60].

An analysis of the relationship between R2D:4D and athletic performance revealed negative correlations ranging from approximately −0.4 to −0.6 in sports such as long-distance running, rowing, rugby, and surfing. However, in sprint and strength events, the associations were found to be weak [48]. These results corroborate our findings, given that powerlifting is a strength-focused sport.

Our study's findings indicate a significant relationship between the R2D:4D and MPV, which is a crucial performance indicator in various sports, particularly those requiring explosive power and muscular strength, such as track and field and swimming [61]. MPV refers to the mean velocity at which the bar is lifted during the concentric phase of a powerlifting movement. This parameter is utilized to assess lifter efficiency and power output and is critical for maximizing performance and minimizing injury risk [62]. Studies have demonstrated a significant relationship between MPV and relative load, enabling coaches to adjust training programs based on bar velocity rather than relying solely on the weight lifted [63].

Considering the physical limitations and specific needs of Paralympic athletes, the importance of MPV in PP is further emphasized by its capacity to monitor fatigue and recovery. Research suggests that MPV can serve as a sensitive indicator of neuromuscular fatigue, assisting coaches in modulating training loads to prevent overexertion and injuries, which are of particular concern for athletes with disabilities [64].

Lastly, we observed a significant positive correlation between the length of the left ring finger (L2) and power output at 80% of 1RM (power 80%), with a Pearson correlation coefficient of 0.542. This suggests that as the length of the left ring finger increases, power output at 80% of 1RM also increases. This finding aligns with studies associating longer finger lengths with superior performance in activities requiring strength and power [48, 65]. For instance, research has shown that a lower R2D:4D correlates with greater muscular strength and power across diverse populations, including athletes. Studies propose that this ratio may reflect differential exposure to prenatal hormones, such as testosterone, which influence muscular development and force-generating capacity [48, 54]. Moreover, a literature review highlighted that the R2D:4D is associated with characteristics linked to physical performance, including muscular attributes such as power and strength, although the precise mechanisms underlying these relationships require further investigation to be fully elucidated [65]. Furthermore, a study presented important evidence regarding the negative association between R2D:4D and competitiveness [66].

Our study was subject to some limitations regarding the analysis of the relationship between 2D:4D and muscle strength, which would be influenced by the observed heterogeneity [51]. We also mention the potential confounding effect of ethnicity, which has been established as a factor in previous studies, which makes it difficult to examine in our study due to the high degree of population heterogeneity. Thus, ethnicity could not be included as a variable in our analyses [59, 67, 68]. Another limitation concerns individuals with disabilities, where it has been observed that certain pathologies and disabilities can interfere with the R2D:4D [69] and other medical conditions [70]. In addition, the present study has only male individuals, which does not allow generalizing the findings to female individuals. However, although our study had a sample of 16 athletes, we must emphasize that it is a sample of elite athletes at a national level and some at an international level.

Future directions for new studies should include the relationship between performance and the relationship between other strength indicators and even muscle activation and imaging indicators with the D2:D4 ratio, among others.

## 5. Conclusions

The study noted some limitations in using the 2D:4D digit ratio as a predictor of dynamic strength performance in Paralympic powerlifters:

• There was a weak relationship with dynamic strength indicators, suggesting that digit ratio may not be a reliable predictor of dynamic strength performance in this specific population.
• There were only moderate correlations between MPV 45% and right 4D and power 80% and left 2D. There was a moderate negative correlation between MVP 45% with right 2D:4D and with R-L 2D:4D diff.
• Other strength indicators should be investigated for this population, such as muscle activation, other strength indicators, imaging, and even muscle architecture may be of interest.

## Figures and Tables

**Table 1:** Characterization of subjects.

**Variables**	**Values**
Age (years)	30.38 ± 7.22
Body weight (kg)	79.19 ± 20.20
Experience (years)	5.31 ± 0.77
Footprint width (cm)	57.41 ± 13.77
1RM bench press test (kg)	141.63 ± 38.11^a^
1RM/body weight	1.83 ± 0.41^b^
MPV 45% 1RM	1.04 ± 0.16

Abbreviation: MPV 45% 1RM, mean propulsive velocity with 45% of 1 repetition maximum.

^a^All athletes with loads that keep them in the top 10 of their national categories.

^b^Bench press values above 1.4 would be considered elite athletes [37].

**Table 2:** Characterization of subjects, mean ± standard deviation (*X* ± SD), and 95% confidence interval (95% CI).

**Variables**	**X** ± **SD**	**95% CI**
Right second	7.52 ± 0.45	7.28–7.77
Right fourth	7.91 ± 0.49	7.65–8.18
Right 2D:4D	0.95 ± 0.03	0.93–0.97
Left second	7.53 ± 0.38	7.32–7.73
Left fourth	7.88 ± 0.55	7.58–8.18
Left 2D:4D	0.96 ± 0.04	0.94–0.98
Right–left 2D:4D difference	−0.01 ± 0.04	−0.03 to 0.01
MPV 45% (m/s)	1, 04 ± 0.15	0.96–1.12
Vmax 45% (m/s)	1.46 ± 0.17	1.37–1.56
Power 45% (W)	537.21 ± 134.38	463.29–611.21
MPV 80% (m/s)	0.50 ± 0.21	0.38–0.61
Vmax 80% (m/s)	0.74 ± 0.29	0.58–0.90
Power 80% (W)	425.11 ± 174.84	328.90–521.35

*Note:* 2D: length of the second finger; 4D: length of the fourth finger; Power 45%: 45% of 1RM, 80%: 80% of 1RM.

Abbreviations: MPV, mean propulsive velocity; Vmax, maximum velocity.

**Table 3:** Correlation between the different ratios of finger length and dynamic force indicators.

**Variable**	**Right second**	**Right fourth**	**Right 2D:4D**	**Left second**	**Left fourth**	**Left 2D:4D**	**R-L 2D:4D diff.**	**MPV45 (m/s)**	**Vmax45 (m/s)**	**Power45 (W)**	**MPV80 (m/s)**	**Vmax80 (m/s)**	**Power80 (W)**
Right second *p*	1.000												
Right fourth *p*	0.840 0984	1.000											
Right 2D:4D *p*	0.064 0.813	**−0.991** ^**$**^ **< 0.001**	1.000										
Left second *p*	**0.801**⁣^∗^ **< 0.001**	0.008 0.976	**−**0.004 0.990	1.000									
Left fourth *p*	**0.760**⁣^∗^ **0.001**	**−**0.194 0.472	0.182 0.499	**0.868**⁣^∗^ **< 0.001**	1.000								
Left 2D:4D *p*	**−**0.374 0.154	0.384 0.143	**−**0.356 0.176	**−**0.329 0.213	**−0.753**⁣^∗^ **0.001**	1.000							
R-L 2D:4D diff. *p*	0.113 0.677	**−0.985** ^**$**^ **< 0.001**	**0.989** **< 0.001**	0.042 0.877	0.285 0.285	**−**0.490 0.054	1.000						
MPV45 (m/s) *p* *R*^2^	0.094 0.729	**0.551** ^**#**^ **0.027** **0.30**	**−0.595** **0.015** **0.35**	0.350 0.184	0.162 0.550	0.155 0.568	**−0.585** ^**#**^ **0.017** **0.34**	1.000					
Vmax45 (m/s) *p*	0.187 0.487	0.347 0.188	**−**0.388 0.137	0.488 0.055	0.263 0.325	0.141 0.603	**−**0.392 0.133	**0.936** ^**$**^ **< 0.001**	1.000				
Power45 (W) *p*	0.224 0.404	**−**0.048 0.859	0.016 0.952	0.491 0.054	0.362 0.168	**−**0.042 0.877	0.019 0.945	0.301 0.257	0.314 0.235	1.000			
MPV80 (m/s) *p*	0.164 0.543	0.061 0.822	**−**0.079 0.772	0.290 0.276	0.210 0.436	**−**0.026 0.923	**−**0.077 0.776	**0.565** ^**#**^ **0.023**	**0.600** ^**#**^ **0.014**	**−**0.062 0.820	1.000		
Vmax80 (m/s) *p*	0.187 0.489	0.001 0.999	**−**0.015 0.955	0.292 0.273	0.207 0.441	**−**0.013 0.961	**−**0.020 0.942	**0.501** ^**#**^ **0.048**	**0.555** ^**#**^ **0.026**	**−**0.092 0.735	**0.986** ^**$**^ **< 0.001**	1.000	
Power80 (W) *p* *R*^2^	0.268 0.316	**−**0.114 0.675	0.082 0.762	**0.542** ^**#**^ **0.030** **0.29**	0.379 0.148	**−**0.008 0.978	0.071 0.794	0.489 0.054	**0.578** ^**#**^ **0.019**	**0.558** ^**#**^ **0.025**	**0.711**⁣^∗^ **0.002**	**0.708**⁣^∗^ **0.002**	1.000

*Note:* Values that showed correlations above 0.5 have been highlighted in bold. 2D: length of the second finger; 4D: length of the fourth finger; MPV: mean propulsive velocity, Vmax: maximum velocity, 45: 45% 1RM, 80: 80% 1RM.

^#^ *r* = 0.5–07, moderate correlation.

⁣^∗^*r* = 07–0.9, high correlation.

^$^ *r* > 0.9, very high correlation.

## Data Availability

The data presented in this study are available upon request to the corresponding author.
